# Preclinical studies performed in appropriate models could help identify optimal timing of combined chemotherapy and immunotherapy

**DOI:** 10.3389/fimmu.2023.1236965

**Published:** 2023-09-07

**Authors:** Yani Berckmans, Jolien Ceusters, Ann Vankerckhoven, Roxanne Wouters, Matteo Riva, An Coosemans

**Affiliations:** ^1^ Department of Oncology, Laboratory of Tumor Immunology and Immunotherapy, Leuven Cancer Institute, KU Leuven, Leuven, Belgium; ^2^ Oncoinvent AS, Oslo, Norway; ^3^ Department of Neurosurgery, Centre Hospitalier Universitaire (CHU) UCLouvain Namur, University Hospital of Godinne, Yvoir, Belgium

**Keywords:** immune checkpoint inhibitors, chemotherapy, preclinical models, combination treatment, cancer therapy

## Abstract

Immune checkpoint inhibitors (ICI) have been revolutionary in the field of cancer therapy. However, their success is limited to specific indications and cancer types. Recently, the combination treatment of ICI and chemotherapy has gained more attention to overcome this limitation. Unfortunately, many clinical trials testing these combinations have provided limited success. This can partly be attributed to an inadequate choice of preclinical models and the lack of scientific rationale to select the most effective immune-oncological combination. In this review, we have analyzed the existing preclinical evidence on this topic, which is only limitedly available. Furthermore, this preclinical data indicates that besides the selection of a specific drug and dose, also the sequence or order of the combination treatment influences the study outcome. Therefore, we conclude that the success of clinical combination trials could be enhanced by improving the preclinical set up, in order to identify the optimal treatment combination and schedule to enhance the anti-tumor immunity.

## 1 Rise in combination therapies with immune checkpoint inhibitors after limited effectivity in monotherapy

In recent years, attention towards cancer immunotherapy has grown (1, 2). The goal is to shift the balance from tumor promoting immunosuppression towards anti-tumor immune activation, to support and promote the immune-mediated attack of cancer cells. Immune checkpoint inhibitors (ICI) are an example of such immunotherapeutic modality aiming to release the tumor induced immune brakes (3). Many of these brakes, or checkpoint molecules, have been identified, such as programmed death ligand 1 (PD-1) or its ligand PD L1, cytotoxic T-lymphocyte associated protein 4 (CTLA-4), T-cell immunoglobin and mucin domain 3 (TIM 3), lymphocyte activation gene 3 (LAG-3) and others (4). Seven such ICIs are currently approved for use in patients across all cancer types. The first approved was the anti-CTLA-4 ipilimumab followed by three anti-PD-1 ICI, nivolumab, cemiplimab and pembrolizumab, and three anti-PD-L1 inhibitors, durvalumab, avelumab and atezolizumab (4, 5). However, as the efficacy of these ICI was mostly observed in a restricted subset of patients, their use is limited to specific indications in cancers such as melanoma, breast cancer, colorectal, classical Hodgkin’s lymphoma and non-small cell lung cancer (NSCLC) (4, 6). In an estimated 60-70% of patients, they remain ineffective (3, 6). To overcome this clinical challenge, attention for combination therapies has grown. In 2018, the clinical research program of the Cancer Research Institute (Anna-Maria Kellen Clinical Accelerator, NY, USA) published an overview of immune-oncological trials, describing the rise in combination trials using PD-1/L1 targeting agents with other immunotherapies, radiotherapy and chemotherapies over the past five years. From the 1105 reported combination studies, the most prevalent combinations included anti-PD-1/L1 with either anti-CTLA-4 (n=251) or chemotherapy (n=170) (7). An update in 2021 showed the continued increase in the number of trials testing a combination of anti-PD-1/L1 and chemotherapy with 145 new trials started in the first three quarters of 2020 alone (8).

## 2 Rationale for combination of chemotherapy with immune checkpoint inhibitors

To develop combination treatments, not only oncological but also immunological changes should be considered. Galluzzi et al. described different immune effects induced by different types of chemotherapy (9). In the past, some chemotherapeutics have been described as immunosuppressive agents, which explained their use for the treatment of severe autoimmune diseases (10). Examples of such chemotherapeutics are cyclophosphamide and methotrexate, which can both impair the proliferation and function of effector T-cells (10, 11). Moreover, doxorubicin has been shown to increase the expansion of myeloid derived suppressor cells, resulting in increased immunosuppression (11). The immense chemotherapeutically induced release of tumor-associated antigens has also been suggested to suppress the anti-tumor immune response. However, evidence supporting this mechanism is currently not available (10). In contrast, cytotoxic chemotherapies have also been reported to promote immunogenicity. For example, one of the immune effects reported for doxorubicin was the enhanced proliferation of tumor targeting CD8+ T-cells, while other conventional chemotherapies like gemcitabine and paclitaxel, were shown to decrease immunosuppressive myeloid derived suppressor cells and regulatory T-cells cells, respectively (9). Similar immunological effects have been discussed in a review by Brown et al., such as the increase of antigen presentation through the upregulation of MHC class 1 on tumor cells by a number of the DNA-damaging therapeutics including gemcitabine, cyclophosphamide and oxaliplatin (12). Our group has shown that carboplatin-paclitaxel resulted in a superior immune-composition compared to several other chemotherapeutics in an ovarian cancer mouse model (13), which was in line with observations in patients (14). Another argument in favor of combining chemotherapy and ICI therapy has been the upregulation of co-inhibitory ligands, such as PD-L1, promoted by numerous chemotherapeutics including paclitaxel, 5-fluorouracil and cisplatin (15). Furthermore, certain chemotherapeutics such as doxorubicin, oxaliplatin and cyclophosphamide could also induce immunogenic cell death (ICD) (12, 16). All together, these immune-related effect of chemotherapy promote the rationale for a combination with ICI treatment to further stimulate the anti-tumor adaptive immune response and T-cell expansion (6, 15). However, all described immune effects can be drug, dose and time dependent, highlighting the importance of optimal knowledge and preclinical evidence (16).

## 3 Combinations of immune checkpoint inhibitors and chemotherapy still fail in the majority of clinical trials

Combinatorial approaches resulted in selected clinical successes (17). In the AtezoTRIBE trial (NCT03721653), previously untreated metastatic colorectal cancer patients showed improved median PFS (HR 0,69; 80% CI [0,56–0,85]) with the addition of the ICI atezolizumab to the treatment schedule consisting of chemotherapy (FOLFOXIRI, a combination of folinic acid, fluorouracil, oxaliplatin and irinotecan) and anti-VEGF (bevacizumab), with all treatments being administered simultaneously using a 48h intravenous infusion with cyclic repeat every 14 days (18). The KEYNOTE-021 trial (NCT02039674) showed improved objective response rate (ORR) (p=0,0016) in NSCLC patients receiving carboplatin/pemetrexed combined with pembrolizumab compared to chemotherapy alone (19). These findings were confirmed in the KEYNOTE-189 trial (NCT02578680) in which overall survival (OS) (HR 0,49; 95% CI [0,38-0,64]) and progression free survival (PFS) (HR 0,52; 95% CI [0,43-0,64]) were significantly increased in the combination therapy-receiving patients (20). Both KEYNOTE-021 and KEYNOTE-189 trials administered pembrolizumab simultaneously with platin-based chemotherapy and pemetrexed for four cycles, repeated every three weeks, as well as maintenance therapy for up to 24 months in combination with pemetrexed (19, 20). Additionally, in patients with metastatic triple negative breast cancer (TNBC) testing positive for PD-L1 expression, the combination of atezolizumab with the chemotherapeutic nab-paclitaxel showed promising results on PFS (HR 0,62; 95% CI [0,49-0,78]) and OS (HR 0,67; 95% CI [0,53-0,86]) (IMpassion130; NCT02425891) when administered simultaneously through intravenous injection on day 1 and 15 of a 28-day cycle which was repeated until disease progression was observed (21). Similar valuable outcomes were observed in the KEYNOTE-522 trial (NCT03036488), where addition of pembrolizumab to neoadjuvant carboplatin paclitaxel chemotherapy yielded a greater response compared to chemotherapy alone in early TNBC patients (HR 0,63; 95% CI [0,43-0,93]) (22).

However, the majority of trials testing an immune-oncological combination produced disappointing results (17). For example, in gastroesophageal cancer patients, the combination of pembrolizumab with chemotherapy (cisplatin and fluorouracil/capecitabine) resulted in a nearly equal OS compared to chemotherapy alone (HR 0,85; 95% CI [0,70-1,03]) in the phase 3 KEYNOTE-062 trial (NCT02494583) (23). Similarly, in the KEYNOTE-361 trial (NCT02853305), no improved PFS (HR 0,78; 95% CI [0,65-0,95]) or OS (HR 0,86; 95% CI [0,72-1,02]) was reported after combination treatment with pembrolizumab and chemotherapy (comprising of gemcitabine and cisplatin/carboplatin) compared to chemotherapy alone, for the treatment of advanced urothelial carcinoma. In this trial, the ICI and chemotherapy were administered simultaneously through intravenous injection on day 1 of a three- weekly cycle with a maximum of six chemotherapy cycles and 35 cycles of pembrolizumab (24). For ovarian cancer, three large phase 3 trials have been published, all reporting negative results. The phase 3 JAVELIN Ovarian 200 trial (NCT02580058) reported no improved response between groups receiving either avelumab (every two weeks, intravenously) combined with pegylated liposomal doxorubicin (PLD) (every four weeks, intravenously) or PLD monotherapy (PFS: HR 0,78; CI [0,59-1,24]; OS: HR 0,89: CI [0,74-1;24]) (25, 26). Likewise, in the JAVELIN Ovarian 100 trial (NCT02718417), the primary objectives of increasing the PFS were not met. In this study, two combination regimens were compared to the control group receiving carboplatin paclitaxel chemotherapy alone; chemotherapy followed by avelumab (HR 1,43; 95% CI [1,05-1,95]) and chemotherapy plus avelumab followed by avelumab (HR 1,14; 95% CI [0,83-1,56]). Remarkably, both combination regimens seemed to perform worse than chemotherapy alone (27, 28). Interim analysis of the IMagyn050 trial (NCT03038100) in newly diagnosed ovarian cancer patients underscored this, as no difference in PFS was seen after addition of atezolizumab to chemotherapy (carboplatin paclitaxel) followed by anti-VEGF (bevacizumab) (HR 0,92; 95% CI [0,97-1,07]). All treatments were administered intravenously on day 1 of a three- weekly cycle. Additionally, both adjuvant and neoadjuvant schedules were included in this trial. However, no discrepancies have been reported between the two groups (29).

## 4 Limited relevant preclinical research could relate to failing clinical trials

**Figure 1:**
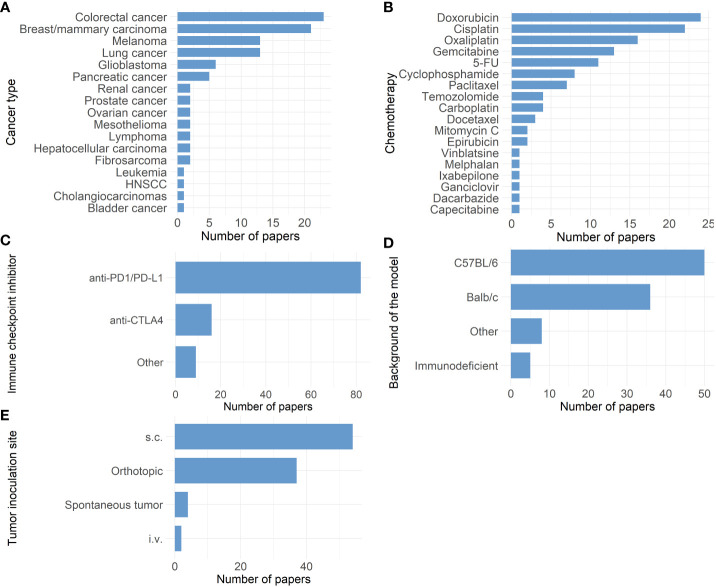
Overview of 92 research papers in which a combination of immune checkpoint inhibitors and chemotherapy was tested preclinically. **(A)** Bar chart showing the different cancer types studied. **(B)** Bar chart showing the distribution of different chemotherapies tested. (5-FU: 5-fluorouracil) **(C)** Bar chart showing the distribution of different immune-checkpoint inhibitors tested. (Other: anti-B7-H3, anti-CD47, anti-CD96, anti-4-1BB, anti-40 and anti-HLA-G) **(D)** Bar chart showing the background of different mouse models used. (Other: C3H, CBA/CaJ, DBA/2J, FVB, KRAS LSL- G12D+; p53-/-: LSL, 129S1/SvImJ, B10.D2, µMt) .**(E)** Bar chart showing the distribution of inoculation sites in the mouse models used. (s.c.: subcutaneous, i.v.: intravenous).

The large number of failed clinical trials testing the combination of ICI with chemotherapy is alarming. The question arises if this could have been anticipated using relevant preclinical research. We performed a PubMed literature search using the minimal search term (chemotherapy [Title/Abstract] AND checkpoint[Title/Abstract] NOT (Review[Publication Type]). This search resulted in 2863 articles on November 12^th^ 2021 of which only 92 research articles were dedicated to preclinical testing of ICI and chemotherapy combinations (See **Supplementary Material 1**). **Figure 1** gives an overview of all different cancer types used in these research articles. Remarkably, almost half (47.8%) of these articles study either colorectal cancer or breast cancer, in which ICI are already accepted for use under specific conditions. More specifically, the U.S. Food and Drug Administration (FDA) approved both pembrolizumab as first line treatment, as well as the combination of ipilimumab and nivolumab in second line treatment of metastatic colorectal cancer in specific conditions. For breast cancer, both atezolizumab and pembrolizumab have been approved for metastatic TNBC patients (4). Although preclinical results are scarce for the majority of other cancer types, clinical trials testing this immune-oncological combination are conducted in a wide range of disease profiles.

**Figures 1B, C** display the different chemotherapeutics and ICI tested in these preclinical studies, respectively. A wide variety of chemotherapeutics have been tested, which can be explained by the different cancer types that were studied and their standard of care in the clinic. In contrast, only two types of ICI prevail: anti-PD-1/L1 and anti-CTLA-4 checkpoint inhibitors. Other ICI modalities are scarcely represented in these preclinical studies. Generally, the choice of preclinical model should be looked at critically before each experiment. Besides the pathogenesis of the disease which should be properly reproduced, it is equally crucial to assure that the biological target of the tested therapy is conserved in the chosen model (30). Therefore, when testing ICI therapy, it is most reasonable to choose an immune competent preclinical model in which the engagement of the ICI with its target on immune cells can be ensured. In the majority of the 92 articles, preclinical research was performed in syngeneic mouse models with C57BL/6 or Balb/c background (**Figure 1D**). Both C57BL/6 and Balb/c are inbred immune competent mice strains. Some inter-strain immunological differences are present, consisting in a prevailing humoral immune response in Balb/c mice, while in C57Bl/6 murine cellular immunity dominates (31). Additionally in other studies, C57Bl/6 mice appeared to have a Th1 dominant immune response in contrast to Balb/c mice where Th2 dominated the immune response (32). Besides these discrepancies, syngeneic models are overall characterized by a fully functioning immune system, which is their main advantage besides their uncomplicated establishment and fair cost. In contrast, five studies could be identified using immunodeficient mouse strains. The use of immunodeficient mouse strains to create models such as patient derived xenografts (PDX) has been proven successful as a preclinical platform (33) and has shown to accurately reflect the gene expression profile and histology of the primary human tumor (34). However, PDX models do not recapitulate the full physiological biology of the human tissue due to the lack of an intact immune system making them less suitable for testing immunotherapeutic strategies (35). Additionally, the response to chemotherapy can also depend on the presence of a fully functioning immune system. In a study by Kroemer and Galluzzi et al., chemotherapy response was shown to be suboptimal in immunodeficient mice compared to syngeneic tumor-bearing mice. This discrepancy could be related to the lack of immunogenic cell death and activation of the subsequent anti-tumor immune response (36). Humanization of these mice created a strategy to overcome the immune limitations seen in immunodeficient xenograft models (37). Different methods for creating humanized mouse models exist such as transplantation of human peripheral blood mononuclear cells or human hematopoietic stem cells into immunodeficient mice after which they can be inoculated using human tumor cells or patient derived tissue (37). This offers an interesting approach to test immunotherapies in models bearing human tumors in combination with a humanized immune system. Multiple caveats however still need to be overcome, such as the development of graft versus host disease and the high cost.

While currently the syngeneic immune competent mouse models are explored most in immune-oncology research, it can be argued that in order to mimic heterogeneous and progressive cancers *in vivo*, genetically engineered mouse models offer added physiological relevance (37). Genetically engineered mouse models carry altered oncogenes and/or tumor suppressor genes and can therefore develop tumor spontaneously (38). Advantages include the orthotopic origin of the tumor with physiological intra-tumoral heterogeneity and an intact immune system, while the extensive variability in tumor development, low mutational burden and the time-consuming process are the largest disadvantages (35, 37). An example of such an established spontaneous cancer model are KPC mice (KrasLSL-G12D+;Trp53LSL-R172H/+;p48Cre in C57Bl/6 mice) which stochastically develop intra-epithelial neoplasms in the pancreas (39). These types of mouse models were only present in four out of the 92 research articles, including models for breast cancer, head and neck squamous cell carcinoma and lung cancer (See **Supplementary Material 1**; Martinez-Usatorre et al., Sci Transl Med, 2021; Sirait-Fischer et al., Front Oncol, 2020; Spielbauer et al., Otolaryngology–Head and Neck Surgery, 2018; Workenhe et al., Commun Biol, 2020).

Lastly, though none of the 92 articles found in our literature search deferred from using mice, in preclinical immune-oncological research, other species are increasingly being considered for use due to their similar immunological, anatomical or pathological features to humans. Syrian hamsters are viewed as ideal preclinical animal model for cancer immunotherapy studies due to the complex tumor microenvironment, tumor histology and cancer progression resembling the human scenario (40). Another advantage of this species are the cheek pouches which are considered an immune desert due to the lack of lymphatic drainage pathways offering the potential of long-term transplantation of foreign material, such as patient derived tumor tissue without immune rejection (40). Secondly, ferrets can be suitable models as they have a particularly high cancer prevalence, which could give insights into the biological development of cancer, and have shown useful in immunology studies (41, 42). Higher animal models such as canines and swine are also available. Canines can spontaneously develop tumors, showing large homology to human cancers both genetically and in relation to the surrounding tumor immune microenvironment (43). They are fully immune competent and possess more comparable immune constituents to the human immune system compared to murine models (43, 44). Swine models can be applied in oncological research and are mostly used to test new devices and surgical procedures (45). Immunological applications can also be explored as swine are genetically and immunologically relevant to humans and have shown to respond similarly to anti-cancer drugs (46). Different models exist such as genetically modified swine capable of developing tumors and wild-type immune competent swine bearing xenograft tumors (45). Lower models such as zebrafish or Xenopus could also provide opportunities to study certain therapeutic effects on cancer cells depending on the research question (44). Homologous to humans, zebrafish possess two branches in their immune system, the innate and the adaptive component. However, one difference is the delay in adaptive immunity development causing the zebrafish to rely solely on the innate immunity in the first stages of their lives. This provides the opportunity for specific studies into the innate immune system using recently hatched (72 hours post fertilization) zebrafish which harbor a fundamentally similar macrophage lineage to humans. At this stage, inoculation of human cancer cells has an increased engraftment chance due to the absence of adaptive immune cells (47). In later stages of zebrafish development, immune-oncological research may be limited due to their biological and anatomical differences, such as the lack of lymph nodes, and less complex immune system compared to humans (44).

Besides animal models, alternative methods for preclinical research are being explored, which is increasingly promoted by governmental and ethical services, but has proven difficult when the immune system is involved. One hopeful approach is the use of *ex vivo*, three dimensional cultures derived from human tumor tissue called explants (48, 49). This methodology has already been successful for multiple cancer types. Moreover, the explant culturing method for NSLC by Karekla et al. showed reproducible drug responses to cisplatin (49). Furthermore, these types of cultures have the potential to provide a platform for evaluating immunological responses to ICI therapy, as reported by Voabil and colleagues (50).

Another point of attention when working with animal models for the purpose of translational research is the fact that the tumor inoculation site can impact the tumor immune environment and subsequently the immunotherapy response (51). Most of these 92 studies use subcutaneous (s.c.) mouse models (**Figure 1E**). Subcutaneously mimicking a tumor that from origin grows in another organ is less ideal as the invasive potential of the tumor is limited due to the presence of s.c. connective tissue, therefore creating a different tumor microenvironment (most likely also influencing the function/role of tissue resident immune cells) (52). This can impact the translatability of these models for immunotherapeutic research. For example, in preclinical melanoma models the tumor location was shown to influence the recruitment of tissue-specific tumor-associated macrophages (51, 52). Orthotopically inoculated tumors in immune-competent mice can surmount some of these limitations. Overall, it seems that the chosen model and inoculation site can impact the outcome in these preclinical studies.

## 5 The therapeutic combinatorial schedule influences treatment response

**Figure 2:**
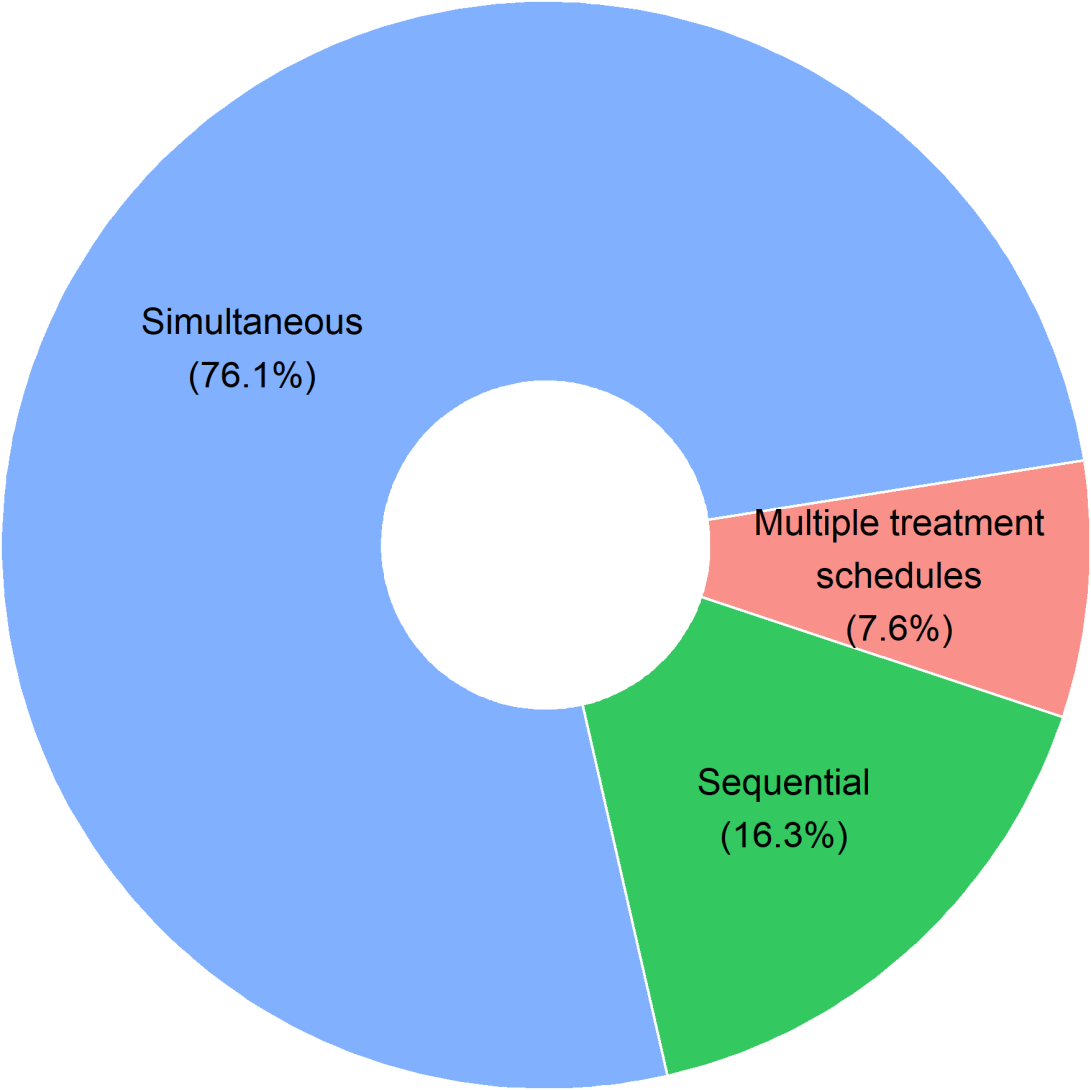
Pie chart on administration schedules in 92 preclinical research articles. Studies with simultaneous treatment administration include all studies with same start date of both therapies (47/92), studies where immune-checkpoint inhibitor treatment started first (6/92) or chemotherapeutic treatment started first (17/92) before simultaneous administration of the combination treatment was continued. Sequential treatment administration includes studies where no overlap between both treatments occurred. Treatments were given consecutively with either first chemotherapy (14/92) or first immune-checkpoint inhibitor therapy (1/92). In 7 out of the 92 studies (7.6%), multiple treatment administration schedules were tested in one preclinical experiment.

Our group has previously described the shift in survival in a syngeneic orthotopic mouse model for ovarian cancer when changing the sequence of the chemotherapy and immunotherapy combination (53). To identify the optimal immune-oncological combination regimen, we looked into the different administration schedules of the 92 preclinical studies (**Figure 2**). In the majority (76,1%; 70/92), administration of ICI and chemotherapy was simultaneous, mostly even started the same day (47/92). Only 16,3% (15/92) of preclinical studies were designed with a sequential administration of treatment, nearly all of them starting with chemotherapy prior to ICI treatment (14/15). Only a small percentage of studies (7,6%; 7/92) investigated multiple different administration schedules. These seven articles will be further discussed below. Additionally, two preclinical studies not identified through our search strategy, but similarly looking into the impact of combination treatment scheduling, have been added manually to the discussion below. An overview of all nine studies can be found in **Table 1**.

**Table 1:** Overview of different combinations of chemotherapy and ICI used in nine research articles comparing multiple therapeutic regimens.

Author – Year	Cancer type	ICI	Chemo-therapy	Regimen of administration tested	Superior regimen	Limitations
**Lesterhuis WJ. et al. – 2013** (54)	Meso-thelioma	anti-CTLA4	Gemcitabine	• Simultaneous • Sequential – first chemo • Sequential – first ICI	Simultaneous	s.c. mouse model
**Martin-Ruiz A. et al. – 2020** (55)	Lung carcinoma	anti-PD-1	Cisplatin	• Simultaneous • Sequential – first chemo • Sequential – first ICI	Simultaneous	Immuno-deficient mouse model (NSG)
**Zhao X. et al. – 2020** (56)	Colorectal carcinoma	anti-PD-1	5-fluorouracil	• Simultaneous • Sequential – first chemo	Sequential – first chemo	s.c. mouse model
**Fu D. et al. – 2020** (57)	Colorectal carcinoma	anti-PD-1/L1	Cisplatin or Oxaliplatin	• Simultaneous • Sequential – first chemo, 3 days later ICI • Sequential first – chemo, 6 days later ICI	Sequential – first chemo, 3 days later ICI	s.c. mouse model
**Golchin S. et al. – 2019** (58)	Colorectal carcinoma	anti-PD-L1	Oxaliplatin	• Simultaneous • Sequential – first chemo • Sequential – first ICI	Sequential – first ICI	s.c. mouse model
**Alimohammadi R. et al. – 2020** (59)	Melanoma	anti-CTLA-4	Doxel	• Simultaneous • Sequential – first chemo • Sequential – first ICI	Sequential – first ICI	/
**Yan Y. et al. – 2018** (60)	Melanoma	anti-PD-1/L1	Carboplatin + Paclitaxel	• Simultaneous • First ICI followed by simultaneous ICI + chemo	First ICI followed by simultaneous ICI + chemo	/
**Coosemans et al. – 2019** (53)	Ovarian cancer	anti-PD-1	Carboplatin + Paclitaxel	• Simultaneous – first ICI followed by ICI + chemo • Simultaneous – first chemo followed by chemo + ICI	Simultaneous – first ICI followed by ICI + chemo	/
**Riva et al. – 2020** (61)	High-Grade Glioma	anti-PD-1	Temozolomide	• Simultaneous • Sequential – first chemo • Sequential – first ICI	No difference	/

(ICI, immune checkpoint inhibitor; s.c., subcutaneous; NSG, NOD scid gamma).

Lesterhuis et al. studied the combination of anti-CTLA-4 and gemcitabine chemotherapy in a s.c. mesothelioma mouse model. A significant survival benefit was reported in mice receiving simultaneous administration of both treatments compared to the regimen where gemcitabine was administered either before or after anti-CTLA-4, although no (immunological) explanation was provided or discussed (54). One other study similarly observed reduced tumor growth when anti-PD-1 was given simultaneously with cisplatin compared to both sequential regimens. Of note, the authors used an immunodeficient mouse model (patient derived xenograft, NOS-SCID gamma mouse) for lung cancer, restricting the reliability of the immune response (55).

Sequential administration of 5-fluorouracil chemotherapy followed by anti-PD-1 appeared to be the best treatment schedule compared to simultaneous administration of both treatments in colorectal cancer. According to this study by Zhao et al., sequential administration of both treatments resulted in increased frequencies of total tumor infiltrated T-cells, as well as the increased expression of PD-L1 on tumor cells. From this, it was suggested that the survival benefit of this combination regimen could be related to the optimal scheduling of the individual immunological effects (56). In accordance to this, another study in colorectal cancer by Fu et al. reported an increased survival when the ICI anti-PD-1/L1 was administered with a specific delay of three days after platin-based chemotherapy compared to simultaneous administration. However, increasing the delay to six days abrogated this beneficial outcome. Subsequent flow cytometric analysis of tumor infiltrating lymphocytes showed an increase of PD-1 positive T cells three days after chemotherapy, followed by a decrease on day seven. This immunological evidence could relate to the narrow window for optimal treatment scheduling (57). In contrast, in a similar s.c. colorectal mouse model, sequential treatment with first anti-PD-L1 followed by oxaliplatin was identified as the most promising treatment regimen compared to both simultaneous treatment or chemotherapy followed by ICI administration (non-significant). This result could be explained by the more pronounced influx of CD8+ T-cells observed in this specific treatment regimen compared to other regimen in this study (58). The discrepancy between the results of the two latter studies could be attributed to the use of different mouse models, MC38 cells in C57BL/6 mice and CT26 cells in Balb/c mice, respectively. As described above, the choice of the preclinical model can influence the immune response and subsequent study outcome.

The sequential schedule in which the ICI was administered prior to chemotherapy, was demonstrated to be superior in two studies performed in orthotopic melanoma mouse models. Both studies showed a rise in CD8+ T-cells with this treatment schedule (59, 60). In the first study, the combination of anti-CTLA-4 followed by Doxil (a liposomal doxorubicin) was superior to both simultaneous and sequential administration in the reverse order (59). Likewise, significant increase in survival was noted in the second melanoma study testing the combination regimen where anti-PD-L1 was administered before carboplatin-paclitaxel compared to simultaneous start of both therapies (60).

It is important to note that in all these research articles, mainly tumor infiltrating effector CD8+ T-cells were evaluated. Analysis of other therapeutically induced immune effects such as influence on the innate immune system or the stimulation of ICD was underrepresented.

As previously mentioned, our group also tested different immune-oncological combination regimens in a syngeneic orthotopic ID8-fLuc ovarian cancer mouse model using a survival analysis. The most beneficial schedule was identified as anti-PD-1 followed by simultaneous carboplatin/paclitaxel chemotherapy. Of note, this combination did not show significant improved survival compared to mice receiving chemotherapy alone. However, inferior results were observed when chemotherapy was started prior to the addition of the ICI therapy (53). Additionally, our group studied different schedules combining (for the first time at the preclinical level) anti-PD-1 with chemotherapy-radiotherapy in an orthotopic high-grade glioma mouse model. However, we did not observe any significant difference, nor in survival, nor in immune composition, between either simultaneous or sequential administration (61).

It is clear from these nine articles (**Table 1**), although they have some limitations, that the order/sequence of treatments can influence the tumor growth and survival preclinically. On the other hand, clinically tested treatment schedules and doses will always differ from those given to preclinical models due to the large variability in treatment options depending on tumor type and stage of cancer patients, compared to animals. This is an unavoidable limitation due to the inherent biological difference between humans and animals. Nevertheless, the influence on the immune system can be identified and extrapolated. For example, positive preclinical results with CTLA-4 inhibitors in mice and non-human primates resulted in the development of the human monoclonal antibody ipilimumab and the following clinical trial successes (62). Additionally, failing clinical trials using combined anti-PD1 and chemotherapy for the treatment of ovarian cancer such as JAVELIN ovarian 100 could be replicated in preclinical studies in an orthotopic syngeneic ovarian cancer mouse model, showing the potential translatability of rationally designed preclinical research (28, 53). P reclinical research is therefore indispensable to ameliorate the outcome of clinical combination trials, in order to determine the optimal sequence for each immune-oncological combination therapy based on their individual immunological effects (recruitment of immune cells, induction of ICD, etc.) and to provide relevant scientific evidence for designing further clinical trials to increase their success rate.

## 6 Conclusion

To conclude, even though a large number of clinical trials testing ICI and chemotherapy combinations have been conducted, a majority of these trials produced disappointing results. A lack of rationally designed preclinical research could partially explain why these trials fail to induce synergy between therapies. Here, preclinical research articles testing this immune-oncological combination treatments were reviewed. Together, the articles discussed in this review show the importance of choosing a relevant model for preclinical research in order to increase the translatability and gather evidence for optimizing the clinical trial design, of preclinical research using the correct preclinical models and of preclinical research evaluating different treatment schedules when combining therapies. Most importantly, combination strategies have to take into consideration the different immunological effects and mechanisms of both the specific chemotherapeutic and ICIs. Increasing the investment into detailed preclinical research focused on identifying the optimal therapeutic regimen, could drastically improve the chance for success in subsequent clinical trials.

## Author contributions

Study concept by AC. Study design by YB. Data analysis and interpretation by YB and JC. Manuscript preparation by YB. Manuscript review by AC, JC, AV, RW, MR. Final manuscript editing by AC. All authors contributed to the article and approved the submitted version.
