# Supplementary material for: Preclinical studies performed in appropriate models could help identify optimal timing of combined chemotherapy and immunotherapy
Source: Front Immunol. 2023 Sep 7;14:1236965. doi: 10.3389/fimmu.2023.1236965 (PMC10512939; doi:10.3389/fimmu.2023.1236965)
Supplement: Supplementary file 1 [file DataSheet_1.docx]

Supplementary Material

Enhancing anti-tumor immunity by optimal timing of combined standard of care chemotherapy and immunotherapy

Berckmans Yani, Ceusters Jolien, Vankerckhoven Ann, Wouters Roxanne, Riva Matteo, Coosemans An^*^

*** Correspondence:** Coosemans An: An.Coosemans@kuleuven.be

# Supplementary Material 1

Full reference list of selected articles:

Ahmad, G., Mackenzie, G. G., Egan, J., & Amiji, M. M. (2019). DHA-SBT-1214 Taxoid Nanoemulsion and Anti-PD-L1 Antibody Combination Therapy Enhances Antitumor Efficacy in a Syngeneic Pancreatic Adenocarcinoma Model. *Molecular Cancer Therapeutics*, *18*(11), 1961–1972. https://doi.org/10.1158/1535-7163.MCT-18-1046

Alimohammadi, R., Alibeigi, R., Nikpoor, A. R., Chalbatani, G. M., Webster, T. J., Jaafari, M. R., & Jalali, S. A. (2020). Encapsulated Checkpoint Blocker Before Chemotherapy: The Optimal Sequence of Anti-CTLA-4 and Doxil Combination Therapy. *International Journal of Nanomedicine*, *15*, 5279–5288. https://doi.org/10.2147/IJN.S260760

Ariyan, C. E., Brady, M. S., Siegelbaum, R. H., Hu, J., Bello, D. M., Rand, J., Fisher, C., Lefkowitz, R. A., Panageas, K. S., Pulitzer, M., Vignali, M., Emerson, R., Tipton, C., Robins, H., Merghoub, T., Yuan, J., Jungbluth, A., Blando, J., Sharma, P., … Allison, J. P. (2018). Robust Antitumor Responses Result from Local Chemotherapy and CTLA-4 Blockade. *Cancer Immunology Research*, *6*(2), 189–200. https://doi.org/10.1158/2326-6066.CIR-17-0356

Aston, W. J., Hope, D. E., Cook, A. M., Boon, L., Dick, I., Nowak, A. K., Lake, R. A., & Lesterhuis, W. J. (n.d.). Dexamethasone differentially depletes tumour and peripheral blood lymphocytes and can impact the efficacy of chemotherapy/checkpoint blockade combination treatment. *Oncoimmunology*, *8*(11), e1641390. https://doi.org/10.1080/2162402X.2019.1641390

Bent, E. H., Millán-Barea, L. R., Zhuang, I., Goulet, D. R., Fröse, J., & Hemann, M. T. (2021). Microenvironmental IL-6 inhibits anti-cancer immune responses generated by cytotoxic chemotherapy. *Nature Communications*, *12*(1), 6218. https://doi.org/10.1038/s41467-021-26407-4

Black, M., Barsoum, I. B., Truesdell, P., Cotechini, T., Macdonald-Goodfellow, S. K., Petroff, M., Siemens, D. R., Koti, M., Craig, A. W. B., & Graham, C. H. (2016). Activation of the PD-1/PD-L1 immune checkpoint confers tumor cell chemoresistance associated with increased metastasis. *Oncotarget*, *7*(9), 10557–10567. https://doi.org/10.18632/oncotarget.7235

Blake, S. J., Stannard, K., Liu, J., Allen, S., Yong, M. C. R., Mittal, D., Aguilera, A. R., Miles, J. J., Lutzky, V. P., de Andrade, L. F., Martinet, L., Colonna, M., Takeda, K., Kühnel, F., Gurlevik, E., Bernhardt, G., Teng, M. W. L., & Smyth, M. J. (2016). Suppression of Metastases Using a New Lymphocyte Checkpoint Target for Cancer Immunotherapy. *Cancer Discovery*, *6*(4), 446–459. https://doi.org/10.1158/2159-8290.CD-15-0944

Bloom, A. C., Bender, L. H., Tiwary, S., Pasquet, L., Clark, K., Jiang, T., Xia, Z., Morales-Kastresana, A., Jones, J. C., Walters, I., Terabe, M., & Berzofsky, J. A. (n.d.). Intratumorally delivered formulation, INT230-6, containing potent anticancer agents induces protective T cell immunity and memory. *Oncoimmunology*, *8*(10), e1625687. https://doi.org/10.1080/2162402X.2019.1625687

Brooks, J., Fleischmann-Mundt, B., Woller, N., Niemann, J., Ribback, S., Peters, K., Demir, I. E., Armbrecht, N., Ceyhan, G. O., Manns, M. P., Wirth, T. C., Kubicka, S., Bernhardt, G., Smyth, M. J., Calvisi, D. F., Gürlevik, E., & Kühnel, F. (2018). Perioperative, Spatiotemporally Coordinated Activation of T and NK Cells Prevents Recurrence of Pancreatic Cancer. *Cancer Research*, *78*(2), 475–488. https://doi.org/10.1158/0008-5472.CAN-17-2415

Cheng, N., Bei, Y., Song, Y., Zhang, W., Xu, L., Zhang, W., Yang, N., Bai, X., Shu, Y., & Shen, P. (2021). B7-H3 augments the pro-angiogenic function of tumor-associated macrophages and acts as a novel adjuvant target for triple-negative breast cancer therapy. *Biochemical Pharmacology*, *183*, 114298. https://doi.org/10.1016/j.bcp.2020.114298

Choi, J., Shim, M. K., Yang, S., Hwang, H. S., Cho, H., Kim, J., Yun, W. S., Moon, Y., Kim, J., Yoon, H. Y., & Kim, K. (2021). Visible-Light-Triggered Prodrug Nanoparticles Combine Chemotherapy and Photodynamic Therapy to Potentiate Checkpoint Blockade Cancer Immunotherapy. *ACS Nano*. https://doi.org/10.1021/acsnano.1c03416

Cubas, R., Moskalenko, M., Cheung, J., Yang, M., McNamara, E., Xiong, H., Hoves, S., Ries, C. H., Kim, J., & Gould, S. (2018). Chemotherapy Combines Effectively with Anti-PD-L1 Treatment and Can Augment Antitumor Responses. *Journal of Immunology (Baltimore, Md. : 1950)*, *201*(8), 2273–2286. https://doi.org/10.4049/jimmunol.1800275

Cui, S. (2017). Immunogenic Chemotherapy Sensitizes Renal Cancer to Immune Checkpoint Blockade Therapy in Preclinical Models. *Medical Science Monitor : International Medical Journal of Experimental and Clinical Research*, *23*, 3360–3366. https://doi.org/10.12659/msm.902426

Diggs, L. P., Ruf, B., Ma, C., Heinrich, B., Cui, L., Zhang, Q., McVey, J. C., Wabitsch, S., Heinrich, S., Rosato, U., Lai, W., Subramanyam, V., Longerich, T., Loosen, S. H., Luedde, T., Neumann, U. P., Desar, S., Kleiner, D., Gores, G., … Greten, T. F. (2021). CD40-mediated immune cell activation enhances response to anti-PD-1 in murine intrahepatic cholangiocarcinoma. *Journal of Hepatology*, *74*(5), 1145–1154. https://doi.org/10.1016/j.jhep.2020.11.037

Do, A.-V., Geary, S. M., Seol, D., Tobias, P., Carlsen, D., Leelakanok, N., Martin, J. A., & Salem, A. K. (2018). Combining ultrasound and intratumoral administration of doxorubicin-loaded microspheres to enhance tumor cell killing. *International Journal of Pharmaceutics*, *539*(1–2), 139–146. https://doi.org/10.1016/j.ijpharm.2018.01.028

Dosset, M., Vargas, T. R., Lagrange, A., Boidot, R., Végran, F., Roussey, A., Chalmin, F., Dondaine, L., Paul, C., Lauret Marie-Joseph, E., Martin, F., Ryffel, B., Borg, C., Adotévi, O., Ghiringhelli, F., & Apetoh, L. (n.d.). PD-1/PD-L1 pathway: an adaptive immune resistance mechanism to immunogenic chemotherapy in colorectal cancer. *Oncoimmunology*, *7*(6), e1433981. https://doi.org/10.1080/2162402X.2018.1433981

Du, B., Wen, X., Wang, Y., Lin, M., & Lai, J. (2020). Gemcitabine and checkpoint blockade exhibit synergistic anti-tumor effects in a model of murine lung carcinoma. *International Immunopharmacology*, *86*, 106694. https://doi.org/10.1016/j.intimp.2020.106694

Fournel, L., Wu, Z., Stadler, N., Damotte, D., Lococo, F., Boulle, G., Ségal-Bendirdjian, E., Bobbio, A., Icard, P., Trédaniel, J., Alifano, M., & Forgez, P. (2019). Cisplatin increases PD-L1 expression and optimizes immune check-point blockade in non-small cell lung cancer. *Cancer Letters*, *464*, 5–14. https://doi.org/10.1016/j.canlet.2019.08.005

Fu, D., Wu, J., Lai, J., Liu, Y., Zhou, L., Chen, L., & Zhang, Q. (2020). T cell recruitment triggered by optimal dose platinum compounds contributes to the therapeutic efficacy of sequential PD-1 blockade in a mouse model of colon cancer. *American Journal of Cancer Research*, *10*(2), 473–490.

Gao, F., Zhang, C., Qiu, W.-X., Dong, X., Zheng, D.-W., Wu, W., & Zhang, X.-Z. (2018). PD-1 Blockade for Improving the Antitumor Efficiency of Polymer-Doxorubicin Nanoprodrug. *Small (Weinheim an Der Bergstrasse, Germany)*, *14*(37), e1802403. https://doi.org/10.1002/smll.201802403

Geng, Z., Wang, L., Liu, K., Liu, J., & Tan, W. (2021). Enhancing anti-PD-1 Immunotherapy by Nanomicelles Self-Assembled from Multivalent Aptamer Drug Conjugates. *Angewandte Chemie (International Ed. in English)*, *60*(28), 15459–15465. https://doi.org/10.1002/anie.202102631

Ghaffari, A., Peterson, N., Khalaj, K., Vitkin, N., Robinson, A., Francis, J.-A., & Koti, M. (2018). STING agonist therapy in combination with PD-1 immune checkpoint blockade enhances response to carboplatin chemotherapy in high-grade serous ovarian cancer. *British Journal of Cancer*, *119*(4), 440–449. https://doi.org/10.1038/s41416-018-0188-5

Gholamin, S., Youssef, O. A., Rafat, M., Esparza, R., Kahn, S., Shahin, M., Giaccia, A. J., Graves, E. E., Weissman, I., Mitra, S., & Cheshier, S. H. (2020). Irradiation or temozolomide chemotherapy enhances anti-CD47 treatment of glioblastoma. *Innate Immunity*, *26*(2), 130–137. https://doi.org/10.1177/1753425919876690

Goggi, J. L., Hartimath, S. v, Xuan, T. Y., Khanapur, S., Jieu, B., Chin, H. X., Ramasamy, B., Cheng, P., Rong, T. J., Fong, Y. F., Yuen, T. Y., Msallam, R., Chacko, A.-M., Renia, L., Johannes, C., Hwang, Y. Y., & Robins, E. G. (2021). Granzyme B PET Imaging of Combined Chemotherapy and Immune Checkpoint Inhibitor Therapy in Colon Cancer. *Molecular Imaging and Biology*, *23*(5), 714–723. https://doi.org/10.1007/s11307-021-01596-y

Golchin, S., Alimohammadi, R., Rostami Nejad, M., & Jalali, S. A. (2019). Synergistic antitumor effect of anti-PD-L1 combined with oxaliplatin on a mouse tumor model. *Journal of Cellular Physiology*, *234*(11), 19866–19874. https://doi.org/10.1002/jcp.28585

Grabosch, S., Bulatovic, M., Zeng, F., Ma, T., Zhang, L., Ross, M., Brozick, J., Fang, Y., Tseng, G., Kim, E., Gambotto, A., Elishaev, E., P Edwards, R., & Vlad, A. M. (2019). Cisplatin-induced immune modulation in ovarian cancer mouse models with distinct inflammation profiles. *Oncogene*, *38*(13), 2380–2393. https://doi.org/10.1038/s41388-018-0581-9

Grasselly, C., Denis, M., Bourguignon, A., Talhi, N., Mathe, D., Tourette, A., Serre, L., Jordheim, L. P., Matera, E. L., & Dumontet, C. (2018). The Antitumor Activity of Combinations of Cytotoxic Chemotherapy and Immune Checkpoint Inhibitors Is Model-Dependent. *Frontiers in Immunology*, *9*, 2100. https://doi.org/10.3389/fimmu.2018.02100

Gu, Z., Wang, Q., Shi, Y., Huang, Y., Zhang, J., Zhang, X., & Lin, G. (2018). Nanotechnology-mediated immunochemotherapy combined with docetaxel and PD-L1 antibody increase therapeutic effects and decrease systemic toxicity. *Journal of Controlled Release : Official Journal of the Controlled Release Society*, *286*, 369–380. https://doi.org/10.1016/j.jconrel.2018.08.011

Guan, Y., Kraus, S. G., Quaney, M. J., Daniels, M. A., Mitchem, J. B., & Teixeiro, E. (2020). FOLFOX Chemotherapy Ameliorates CD8 T Lymphocyte Exhaustion and Enhances Checkpoint Blockade Efficacy in Colorectal Cancer. *Frontiers in Oncology*, *10*, 586. https://doi.org/10.3389/fonc.2020.00586

He, X., Du, Y., Wang, Z., Wang, X., Duan, J., Wan, R., Xu, J., Zhang, P., Wang, D., Tian, Y., Han, J., Fei, K., Bai, H., Tian, J., & Wang, J. (2020). Upfront dose-reduced chemotherapy synergizes with immunotherapy to optimize chemoimmunotherapy in squamous cell lung carcinoma. *Journal for Immunotherapy of Cancer*, *8*(2). https://doi.org/10.1136/jitc-2020-000807

Ho, T. T. B., Nasti, A., Seki, A., Komura, T., Inui, H., Kozaka, T., Kitamura, Y., Shiba, K., Yamashita, T., Yamashita, T., Mizukoshi, E., Kawaguchi, K., Wada, T., Honda, M., Kaneko, S., & Sakai, Y. (2020). Combination of gemcitabine and anti-PD-1 antibody enhances the anticancer effect of M1 macrophages and the Th1 response in a murine model of pancreatic cancer liver metastasis. *Journal for Immunotherapy of Cancer*, *8*(2). https://doi.org/10.1136/jitc-2020-001367

Hu, M., Zhang, J., Kong, L., Yu, Y., Hu, Q., Yang, T., Wang, Y., Tu, K., Qiao, Q., Qin, X., & Zhang, Z. (2021). Immunogenic Hybrid Nanovesicles of Liposomes and Tumor-Derived Nanovesicles for Cancer Immunochemotherapy. *ACS Nano*, *15*(2), 3123–3138. https://doi.org/10.1021/acsnano.0c09681

Jan, C.-I., Huang, S.-W., Canoll, P., Bruce, J. N., Lin, Y.-C., Pan, C.-M., Lu, H.-M., Chiu, S.-C., & Cho, D.-Y. (2021). Targeting human leukocyte antigen G with chimeric antigen receptors of natural killer cells convert immunosuppression to ablate solid tumors. *Journal for Immunotherapy of Cancer*, *9*(10). https://doi.org/10.1136/jitc-2021-003050

Jia, L., Pang, M., Fan, M., Tan, X., Wang, Y., Huang, M., Liu, Y., Wang, Q., Zhu, Y., & Yang, X. (2020). A pH-responsive Pickering Nanoemulsion for specified spatial delivery of Immune Checkpoint Inhibitor and Chemotherapy agent to Tumors. *Theranostics*, *10*(22), 9956–9969. https://doi.org/10.7150/thno.46089

Jiang, M., Chen, W., Yu, W., Xu, Z., Liu, X., Jia, Q., Guan, X., & Zhang, W. (2021). Sequentially pH-Responsive Drug-Delivery Nanosystem for Tumor Immunogenic Cell Death and Cooperating with Immune Checkpoint Blockade for Efficient Cancer Chemoimmunotherapy. *ACS Applied Materials & Interfaces*, *13*(37), 43963–43974. https://doi.org/10.1021/acsami.1c10643

Kang, T. H., Park, J. H., Yang, A., Park, H. J., Lee, S. E., Kim, Y. S., Jang, G.-Y., Farmer, E., Lam, B., Park, Y.-M., & Hung, C.-F. (2020). Annexin A5 as an immune checkpoint inhibitor and tumor-homing molecule for cancer treatment. *Nature Communications*, *11*(1), 1137. https://doi.org/10.1038/s41467-020-14821-z

Karachi, A., Yang, C., Dastmalchi, F., Sayour, E. J., Huang, J., Azari, H., Long, Y., Flores, C., Mitchell, D. A., & Rahman, M. (2019). Modulation of temozolomide dose differentially affects T-cell response to immune checkpoint inhibition. *Neuro-Oncology*, *21*(6), 730–741. https://doi.org/10.1093/neuonc/noz015

Kuai, R., Yuan, W., Son, S., Nam, J., Xu, Y., Fan, Y., Schwendeman, A., & Moon, J. J. (2018). Elimination of established tumors with nanodisc-based combination chemoimmunotherapy. *Science Advances*, *4*(4), eaao1736. https://doi.org/10.1126/sciadv.aao1736

Kuczynski, E. A., Krueger, J., Chow, A., Xu, P., Man, S., Sundaravadanam, Y., Miller, J. K., Krzyzanowski, P. M., & Kerbel, R. S. (2018). Impact of Chemical-Induced Mutational Load Increase on Immune Checkpoint Therapy in Poorly Responsive Murine Tumors. *Molecular Cancer Therapeutics*, *17*(4), 869–882. https://doi.org/10.1158/1535-7163.MCT-17-1091

Lan, Y., Zhang, D., Xu, C., Hance, K. W., Marelli, B., Qi, J., Yu, H., Qin, G., Sircar, A., Hernández, V. M., Jenkins, M. H., Fontana, R. E., Deshpande, A., Locke, G., Sabzevari, H., Radvanyi, L., & Lo, K.-M. (2018). Enhanced preclinical antitumor activity of M7824, a bifunctional fusion protein simultaneously targeting PD-L1 and TGF-β. *Science Translational Medicine*, *10*(424). https://doi.org/10.1126/scitranslmed.aan5488

Lauret Marie Joseph, E., Kirilovsky, A., Lecoester, B., el Sissy, C., Boullerot, L., Rangan, L., Marguier, A., Tochet, F., Dosset, M., Boustani, J., Ravel, P., Boidot, R., Spehner, L., Haicheur-Adjouri, N., Marliot, F., Pallandre, J.-R., Bonnefoy, F., Scripcariu, V., van den Eynde, M., … Adotevi, O. (2021). Chemoradiation triggers antitumor Th1 and tissue resident memory-polarized immune responses to improve immune checkpoint inhibitors therapy. *Journal for Immunotherapy of Cancer*, *9*(7). https://doi.org/10.1136/jitc-2020-002256

Lee, E. J., Jang, G.-Y., Lee, S. E., Lee, J. W., Han, H. D., Park, Y.-M., & Kang, T. H. (2021). A novel form of immunotherapy using antigen peptides conjugated on PD-L1 antibody. *Immunology Letters*, *240*, 137–148. https://doi.org/10.1016/j.imlet.2021.10.006

Lévesque, S., le Naour, J., Pietrocola, F., Paillet, J., Kremer, M., Castoldi, F., Baracco, E. E., Wang, Y., Vacchelli, E., Stoll, G., Jolly, A., de La Grange, P., Zitvogel, L., Kroemer, G., & Pol, J. G. (n.d.). A synergistic triad of chemotherapy, immune checkpoint inhibitors, and caloric restriction mimetics eradicates tumors in mice. *Oncoimmunology*, *8*(11), e1657375. https://doi.org/10.1080/2162402X.2019.1657375

Limagne, E., Thibaudin, M., Nuttin, L., Spill, A., Derangère, V., Fumet, J.-D., Amellal, N., Peranzoni, E., Cattan, V., & Ghiringhelli, F. (2019). Trifluridine/Tipiracil plus Oxaliplatin Improves PD-1 Blockade in Colorectal Cancer by Inducing Immunogenic Cell Death and Depleting Macrophages. *Cancer Immunology Research*, *7*(12), 1958–1969. https://doi.org/10.1158/2326-6066.CIR-19-0228

Lu, C.-S., Lin, C.-W., Chang, Y.-H., Chen, H.-Y., Chung, W.-C., Lai, W.-Y., Ho, C.-C., Wang, T.-H., Chen, C.-Y., Yeh, C.-L., Wu, S., Wang, S.-P., & Yang, P.-C. (2020). Antimetabolite pemetrexed primes a favorable tumor microenvironment for immune checkpoint blockade therapy. *Journal for Immunotherapy of Cancer*, *8*(2). https://doi.org/10.1136/jitc-2020-001392

Luo, R., Firat, E., Gaedicke, S., Guffart, E., Watanabe, T., & Niedermann, G. (2019). Cisplatin Facilitates Radiation-Induced Abscopal Effects in Conjunction with PD-1 Checkpoint Blockade Through CXCR3/CXCL10-Mediated T-cell Recruitment. *Clinical Cancer Research : An Official Journal of the American Association for Cancer Research*, *25*(23), 7243–7255. https://doi.org/10.1158/1078-0432.CCR-19-1344

Martinez-Usatorre, A., Kadioglu, E., Boivin, G., Cianciaruso, C., Guichard, A., Torchia, B., Zangger, N., Nassiri, S., Keklikoglou, I., Schmittnaegel, M., Ries, C. H., Meylan, E., & de Palma, M. (2021). Overcoming microenvironmental resistance to PD-1 blockade in genetically engineered lung cancer models. *Science Translational Medicine*, *13*(606). https://doi.org/10.1126/scitranslmed.abd1616

Martín-Ruiz, A., Fiuza-Luces, C., Martínez-Martínez, E., Arias, C. F., Gutiérrez, L., Ramírez, M., Martín-Acosta, P., Coronado, M. J., Lucia, A., & Provencio, M. (2020). Effects of anti-PD-1 immunotherapy on tumor regression: insights from a patient-derived xenograft model. *Scientific Reports*, *10*(1), 7078. https://doi.org/10.1038/s41598-020-63796-w

Pfirschke, C., Engblom, C., Rickelt, S., Cortez-Retamozo, V., Garris, C., Pucci, F., Yamazaki, T., Poirier-Colame, V., Newton, A., Redouane, Y., Lin, Y.-J., Wojtkiewicz, G., Iwamoto, Y., Mino-Kenudson, M., Huynh, T. G., Hynes, R. O., Freeman, G. J., Kroemer, G., Zitvogel, L., … Pittet, M. J. (2016). Immunogenic Chemotherapy Sensitizes Tumors to Checkpoint Blockade Therapy. *Immunity*, *44*(2), 343–354. https://doi.org/10.1016/j.immuni.2015.11.024

Qin, S., Yu, Y., Guan, H., Yang, Y., Sun, F., Sun, Y., Zhu, J., Xing, L., Yu, J., & Sun, X. (2021). A preclinical study: correlation between PD-L1 PET imaging and the prediction of therapy efficacy of MC38 tumor with 68Ga-labeled PD-L1 targeted nanobody. *Aging*, *13*(9), 13006–13022. https://doi.org/10.18632/aging.202981

Reguera-Nuñez, E., Xu, P., Chow, A., Man, S., Hilberg, F., & Kerbel, R. S. (2019). Therapeutic impact of Nintedanib with paclitaxel and/or a PD-L1 antibody in preclinical models of orthotopic primary or metastatic triple negative breast cancer. *Journal of Experimental & Clinical Cancer Research : CR*, *38*(1), 16. https://doi.org/10.1186/s13046-018-0999-5

Reul, J., Frisch, J., Engeland, C. E., Thalheimer, F. B., Hartmann, J., Ungerechts, G., & Buchholz, C. J. (2019). Tumor-Specific Delivery of Immune Checkpoint Inhibitors by Engineered AAV Vectors. *Frontiers in Oncology*, *9*, 52. https://doi.org/10.3389/fonc.2019.00052

Rossi, A., Lucarini, V., Macchia, I., Sestili, P., Buccione, C., Donati, S., Ciccolella, M., Sistigu, A., D’Urso, M. T., Pacca, A. M., Cardarelli, E., Mattei, F., Proietti, E., Schiavoni, G., & Bracci, L. (2020). Tumor-Intrinsic or Drug-Induced Immunogenicity Dictates the Therapeutic Success of the PD1/PDL Axis Blockade. *Cells*, *9*(4). https://doi.org/10.3390/cells9040940

Roux, C., Jafari, S. M., Shinde, R., Duncan, G., Cescon, D. W., Silvester, J., Chu, M. F., Hodgson, K., Berger, T., Wakeham, A., Palomero, L., Garcia-Valero, M., Pujana, M. A., Mak, T. W., McGaha, T. L., Cappello, P., & Gorrini, C. (2019). Reactive oxygen species modulate macrophage immunosuppressive phenotype through the up-regulation of PD-L1. *Proceedings of the National Academy of Sciences of the United States of America*, *116*(10), 4326–4335. https://doi.org/10.1073/pnas.1819473116

Sen, T., della Corte, C. M., Milutinovic, S., Cardnell, R. J., Diao, L., Ramkumar, K., Gay, C. M., Stewart, C. A., Fan, Y., Shen, L., Hansen, R. J., Strouse, B., Hedrick, M. P., Hassig, C. A., Heymach, J. v, Wang, J., & Byers, L. A. (2019). Combination Treatment of the Oral CHK1 Inhibitor, SRA737, and Low-Dose Gemcitabine Enhances the Effect of Programmed Death Ligand 1 Blockade by Modulating the Immune Microenvironment in SCLC. *Journal of Thoracic Oncology : Official Publication of the International Association for the Study of Lung Cancer*, *14*(12), 2152–2163. https://doi.org/10.1016/j.jtho.2019.08.009

Shao, D., Zhang, F., Chen, F., Zheng, X., Hu, H., Yang, C., Tu, Z., Wang, Z., Chang, Z., Lu, J., Li, T., Zhang, Y., Chen, L., Leong, K. W., & Dong, W.-F. (2020). Biomimetic Diselenide-Bridged Mesoporous Organosilica Nanoparticles as an X-ray-Responsive Biodegradable Carrier for Chemo-Immunotherapy. *Advanced Materials (Deerfield Beach, Fla.)*, *32*(50), e2004385. https://doi.org/10.1002/adma.202004385

Shirinbak, S., Chan, R. Y., Shahani, S., Muthugounder, S., Kennedy, R., Hung, L. T., Fernandez, G. E., Hadjidaniel, M. D., Moghimi, B., Sheard, M. A., Epstein, A. L., Fabbri, M., Shimada, H., & Asgharzadeh, S. (2021). Combined immune checkpoint blockade increases CD8+CD28+PD-1+ effector T cells and provides a therapeutic strategy for patients with neuroblastoma. *Oncoimmunology*, *10*(1), 1838140. https://doi.org/10.1080/2162402X.2020.1838140

Si, X., Ji, G., Ma, S., Xu, Y., Zhao, J., Huang, Z., Zhang, Y., Song, W., & Tang, Z. (2020). Biodegradable Implants Combined with Immunogenic Chemotherapy and Immune Checkpoint Therapy for Peritoneal Metastatic Carcinoma Postoperative Treatment. *ACS Biomaterials Science & Engineering*, *6*(9), 5281–5289. https://doi.org/10.1021/acsbiomaterials.0c00840

Sirait-Fischer, E., Olesch, C., Fink, A. F., Berkefeld, M., Huard, A., Schmid, T., Takeda, K., Brüne, B., & Weigert, A. (2020). Immune Checkpoint Blockade Improves Chemotherapy in the PyMT Mammary Carcinoma Mouse Model. *Frontiers in Oncology*, *10*, 1771. https://doi.org/10.3389/fonc.2020.01771

Skavatsou, E., Semitekolou, M., Morianos, I., Karampelas, T., Lougiakis, N., Xanthou, G., & Tamvakopoulos, C. (2021). Immunotherapy Combined with Metronomic Dosing: An Effective Approach for the Treatment of NSCLC. *Cancers*, *13*(8). https://doi.org/10.3390/cancers13081901

Song, W., Shen, L., Wang, Y., Liu, Q., Goodwin, T. J., Li, J., Dorosheva, O., Liu, T., Liu, R., & Huang, L. (2018). Synergistic and low adverse effect cancer immunotherapy by immunogenic chemotherapy and locally expressed PD-L1 trap. *Nature Communications*, *9*(1), 2237. https://doi.org/10.1038/s41467-018-04605-x

Song, X., Zhou, Z., Li, H., Xue, Y., Lu, X., Bahar, I., Kepp, O., Hung, M.-C., Kroemer, G., & Wan, Y. (2020). Pharmacologic Suppression of B7-H4 Glycosylation Restores Antitumor Immunity in Immune-Cold Breast Cancers. *Cancer Discovery*, *10*(12), 1872–1893. https://doi.org/10.1158/2159-8290.CD-20-0402

Speranza, M.-C., Passaro, C., Ricklefs, F., Kasai, K., Klein, S. R., Nakashima, H., Kaufmann, J. K., Ahmed, A.-K., Nowicki, M. O., Obi, P., Bronisz, A., Aguilar-Cordova, E., Aguilar, L. K., Guzik, B. W., Breakefield, X., Weissleder, R., Freeman, G. J., Reardon, D. A., Wen, P. Y., … Lawler, S. E. (2018). Preclinical investigation of combined gene-mediated cytotoxic immunotherapy and immune checkpoint blockade in glioblastoma. *Neuro-Oncology*, *20*(2), 225–235. https://doi.org/10.1093/neuonc/nox139

Spielbauer, K., Cunningham, L., & Schmitt, N. (2018). PD-1 Inhibition Minimally Affects Cisplatin-Induced Toxicities in a Murine Model. *Otolaryngology--Head and Neck Surgery : Official Journal of American Academy of Otolaryngology-Head and Neck Surgery*, *159*(2), 343–346. https://doi.org/10.1177/0194599818767621

Sun, J.-J., Chen, Y.-C., Huang, Y.-X., Zhao, W.-C., Liu, Y.-H., Venkataramanan, R., Lu, B.-F., & Li, S. (2017). Programmable co-delivery of the immune checkpoint inhibitor NLG919 and chemotherapeutic doxorubicin via a redox-responsive immunostimulatory polymeric prodrug carrier. *Acta Pharmacologica Sinica*, *38*(6), 823–834. https://doi.org/10.1038/aps.2017.44

Tagliamonte, M., Mauriello, A., Cavalluzzo, B., Ragone, C., Manolio, C., Luciano, A., Barbieri, A., Palma, G., Scognamiglio, G., di Mauro, A., di Bonito, M., Tornesello, M. L., Buonaguro, F. M., Vitagliano, L., Caporale, A., Ruvo, M., & Buonaguro, L. (2021). MHC-Optimized Peptide Scaffold for Improved Antigen Presentation and Anti-Tumor Response. *Frontiers in Immunology*, *12*, 769799. https://doi.org/10.3389/fimmu.2021.769799

Tallón de Lara, P., Cecconi, V., Hiltbrunner, S., Yagita, H., Friess, M., Bode, B., Opitz, I., Vrugt, B., Weder, W., Stolzmann, P., Felley-Bosco, E., Stahel, R. A., Tischler, V., Britschgi, C., Soldini, D., van den Broek, M., & Curioni-Fontecedro, A. (2018). Gemcitabine Synergizes with Immune Checkpoint Inhibitors and Overcomes Resistance in a Preclinical Model and Mesothelioma Patients. *Clinical Cancer Research : An Official Journal of the American Association for Cancer Research*, *24*(24), 6345–6354. https://doi.org/10.1158/1078-0432.CCR-18-1231

Taniura, T., Iida, Y., Kotani, H., Ishitobi, K., Tajima, Y., & Harada, M. (2020). Immunogenic chemotherapy in two mouse colon cancer models. *Cancer Science*, *111*(10), 3527–3539. https://doi.org/10.1111/cas.14624

Tong, Q.-S., Miao, W.-M., Huang, H., Luo, J.-Q., Liu, R., Huang, Y.-C., Zhao, D.-K., Shen, S., Du, J.-Z., & Wang, J. (2021). A Tumor-Penetrating Nanomedicine Improves the Chemoimmunotherapy of Pancreatic Cancer. *Small (Weinheim an Der Bergstrasse, Germany)*, *17*(29), e2101208. https://doi.org/10.1002/smll.202101208

Vito, A., Salem, O., El-Sayes, N., MacFawn, I. P., Portillo, A. L., Milne, K., Harrington, D., Ashkar, A. A., Wan, Y., Workenhe, S. T., Nelson, B. H., Bruno, T. C., & Mossman, K. L. (2021). Immune checkpoint blockade in triple negative breast cancer influenced by B cells through myeloid-derived suppressor cells. *Communications Biology*, *4*(1), 859. https://doi.org/10.1038/s42003-021-02375-9

Wada, S., Jackson, C. M., Yoshimura, K., Yen, H.-R., Getnet, D., Harris, T. J., Goldberg, M. v, Bruno, T. C., Grosso, J. F., Durham, N., Netto, G. J., Pardoll, D. M., & Drake, C. G. (2013). Sequencing CTLA-4 blockade with cell-based immunotherapy for prostate cancer. *Journal of Translational Medicine*, *11*, 89. https://doi.org/10.1186/1479-5876-11-89

Wang, H., Liu, Y., Zhu, X., Chen, C., Fu, Z., Wang, M., Lin, D., Chen, Z., Lu, C., & Yang, H. (2021). Multistage Cooperative Nanodrug Combined with PD-L1 for Enhancing Antitumor Chemoimmunotherapy. *Advanced Healthcare Materials*, *10*(21), e2101199. https://doi.org/10.1002/adhm.202101199

Wang, W., Wu, L., Zhang, J., Wu, H., Han, E., & Guo, Q. (2017). Chemoimmunotherapy by combining oxaliplatin with immune checkpoint blockades reduced tumor burden in colorectal cancer animal model. *Biochemical and Biophysical Research Communications*, *487*(1), 1–7. https://doi.org/10.1016/j.bbrc.2016.12.180

Wen, Y., Chen, X., Zhu, X., Gong, Y., Yuan, G., Qin, X., & Liu, J. (2019). Photothermal-Chemotherapy Integrated Nanoparticles with Tumor Microenvironment Response Enhanced the Induction of Immunogenic Cell Death for Colorectal Cancer Efficient Treatment. *ACS Applied Materials & Interfaces*, *11*(46), 43393–43408. https://doi.org/10.1021/acsami.9b17137

Winograd, R., Byrne, K. T., Evans, R. A., Odorizzi, P. M., Meyer, A. R. L., Bajor, D. L., Clendenin, C., Stanger, B. Z., Furth, E. E., Wherry, E. J., & Vonderheide, R. H. (2015). Induction of T-cell Immunity Overcomes Complete Resistance to PD-1 and CTLA-4 Blockade and Improves Survival in Pancreatic Carcinoma. *Cancer Immunology Research*, *3*(4), 399–411. https://doi.org/10.1158/2326-6066.CIR-14-0215

Workenhe, S. T., Nguyen, A., Bakhshinyan, D., Wei, J., Hare, D. N., MacNeill, K. L., Wan, Y., Oberst, A., Bramson, J. L., Nasir, J. A., Vito, A., El-Sayes, N., Singh, S. K., McArthur, A. G., & Mossman, K. L. (2020). De novo necroptosis creates an inflammatory environment mediating tumor susceptibility to immune checkpoint inhibitors. *Communications Biology*, *3*(1), 645. https://doi.org/10.1038/s42003-020-01362-w

Wu, J., Chen, J., Feng, Y., Zhang, S., Lin, L., Guo, Z., Sun, P., Xu, C., Tian, H., & Chen, X. (2020). An immune cocktail therapy to realize multiple boosting of the cancer-immunity cycle by combination of drug/gene delivery nanoparticles. *Science Advances*, *6*(40). https://doi.org/10.1126/sciadv.abc7828

Wu, S., Calero-Pérez, P., Arús, C., & Candiota, A. P. (2020). Anti-PD-1 Immunotherapy in Preclinical GL261 Glioblastoma: Influence of Therapeutic Parameters and Non-Invasive Response Biomarker Assessment with MRSI-Based Approaches. *International Journal of Molecular Sciences*, *21*(22). https://doi.org/10.3390/ijms21228775

Wu, X., Li, Y., Liu, X., Chen, C., Harrington, S. M., Cao, S., Xie, T., Pham, T., Mansfield, A. S., Yan, Y., Kwon, E. D., Wang, L., Ling, K., & Dong, H. (2018). Targeting B7-H1 (PD-L1) sensitizes cancer cells to chemotherapy. *Heliyon*, *4*(12), e01039. https://doi.org/10.1016/j.heliyon.2018.e01039

Xu, J., Zheng, Q., Cheng, X., Hu, S., Zhang, C., Zhou, X., Sun, P., Wang, W., Su, Z., Zou, T., Song, Z., Xia, Y., Yi, X., & Gao, Y. (2021). Chemo-photodynamic therapy with light-triggered disassembly of theranostic nanoplatform in combination with checkpoint blockade for immunotherapy of hepatocellular carcinoma. *Journal of Nanobiotechnology*, *19*(1), 355. https://doi.org/10.1186/s12951-021-01101-1

Yan, Y., Cao, S., Liu, X., Harrington, S. M., Bindeman, W. E., Adjei, A. A., Jang, J. S., Jen, J., Li, Y., Chanana, P., Mansfield, A. S., Park, S. S., Markovic, S. N., Dronca, R. S., & Dong, H. (2018). CX3CR1 identifies PD-1 therapy-responsive CD8+ T cells that withstand chemotherapy during cancer chemoimmunotherapy. *JCI Insight*, *3*(8). https://doi.org/10.1172/jci.insight.97828

Yang, S., Shim, M. K., Kim, W. J., Choi, J., Nam, G.-H., Kim, J., Kim, J., Moon, Y., Kim, H. Y., Park, J., Park, Y., Kim, I.-S., Ryu, J. H., & Kim, K. (2021). Cancer-activated doxorubicin prodrug nanoparticles induce preferential immune response with minimal doxorubicin-related toxicity. *Biomaterials*, *272*, 120791. https://doi.org/10.1016/j.biomaterials.2021.120791

Zhang, F., Chen, F., Yang, C., Wang, L., Hu, H., Li, X., Zheng, X., Wang, Z., Chang, Z., Li, T., Li, L., Ge, M., Du, J., Sun, W., Dong, W.-F., & Shao, D. (2021). Coordination and Redox Dual-Responsive Mesoporous Organosilica Nanoparticles Amplify Immunogenic Cell Death for Cancer Chemoimmunotherapy. *Small (Weinheim an Der Bergstrasse, Germany)*, *17*(26), e2100006. https://doi.org/10.1002/smll.202100006

Zhang, R., Lyu, C., Lu, W., Pu, Y., Jiang, Y., & Deng, Q. (2020). Synergistic effect of programmed death-1 inhibitor and programmed death-1 ligand-1 inhibitor combined with chemotherapeutic drugs on DLBCL cell lines in vitro and in vivo. *American Journal of Cancer Research*, *10*(9), 2800–2812.

Zhao, P., Qiu, L., Zhou, S., Li, L., Qian, Z., & Zhang, H. (2021). Cancer Cell Membrane Camouflaged Mesoporous Silica Nanoparticles Combined with Immune Checkpoint Blockade for Regulating Tumor Microenvironment and Enhancing Antitumor Therapy. *International Journal of Nanomedicine*, *16*, 2107–2121. https://doi.org/10.2147/IJN.S295565

Zhao, X., Kassaye, B., Wangmo, D., Lou, E., & Subramanian, S. (2020). Chemotherapy but Not the Tumor Draining Lymph Nodes Determine the Immunotherapy Response in Secondary Tumors. *IScience*, *23*(5), 101056. https://doi.org/10.1016/j.isci.2020.101056

Zhou, C., Dong, X., Song, C., Cui, S., Chen, T., Zhang, D., Zhao, X., & Yang, C. (2021). Rational Design of Hyaluronic Acid-Based Copolymer-Mixed Micelle in Combination PD-L1 Immune Checkpoint Blockade for Enhanced Chemo-Immunotherapy of Melanoma. *Frontiers in Bioengineering and Biotechnology*, *9*, 653417. https://doi.org/10.3389/fbioe.2021.653417
